# Stem cell therapy: how to do it right

**DOI:** 10.3389/fcell.2014.00066

**Published:** 2014-11-06

**Authors:** Lidia Cova, Dan Lindholm

**Affiliations:** ^1^Department of Neurology and Lab. Neuroscience, Istituto di Ricovero e Cura a Carattere Scientifico, Istituto Auxologico ItalianoMilan, Italy; ^2^Institute of Biomedicine/Biochemistry and Developmental Biology, University of HelsinkiHelsinki, Finland; ^3^Minerva Medical Research InstituteHelsinki, Finland

**Keywords:** regenerative medicine, clinical trials as topic, stem cell transplantation, brain diseases, clinical therapy

Stem cell therapies in brain diseases have in recent years met with a mix of both disappointing and encouraging news. The road taken from the early high hopes and great promises for such treatment to the current more sobering experiments have been cumbersome but also highly instructive and rewarding. Thus, new technologies for delivery and for obtaining progenitor cells and specific classes of neurons including bone marrow cells and induced Pluripotent Stem (iPS) cells have been developed and carry with them renewed interests and hopes for stem cell treatments (Banerjee et al., 2014; Inoue et al., 2014). Successful stem cell application in the brain, however, still have some obstacles and drawbacks that are related to the absence of international consensus procedures for stem cell derivation, the characterization and management for their transplantation in clinical trials/animal models of neurodegenerative diseases. More attention paid to these missing links by scientists could positively influence outcome after grafting and enable direct comparisons between studies done in different Institutes.

Along this line the recent paper by Viero et al. (2014) gives a good perspective on the research and clinical management as well as benefits ascribed to stem cells in brain diseases. From their own points of view with large experience on neuronal progenitor cells the authors review current advances in the field and also discuss future directions to improve and manage stem cell therapies in brain diseases. This is very useful and could pave the way for better designed and well-structured clinical trials.

A recent example to mention in this regard is the multi-center, open-label, fetal cell TRANSEURO PD trial in which 150 patients are presently enrolled (http://clinicaltrials.gov/show/NCT01898390). Previous fetal cell transplants in Parkinson's Disease patients (PD) started in 1987 and the results of these have varied from positive outcomes in small open label trials, to mixed results in two double-blind, placebo-controlled clinical trials. This then in 2003 led to a halt with this approach that was followed by a reassessment by scientists and doctors of the potentials and the future of cell transplantations in PD (Inoue et al., 2014). Recent optimization of the methods with major technical improvements in cell derivation and use of alternative stem cell sources including human Embryonic Stem Cells (hESCs), and a careful revision of past trials, appropriate patient selections, tissue placement and design such as done in TRANSEURO PD trial has led to purer concentrations of early differentiated dopamine cells that may be ideal for transplantation (Barker et al., 2013; Bega and Krainc, 2014).

In their paper Viero et al. have summarized their data with two human cell lines, hESCs CCTL14 and the SPC-01 neural stem cell line derived from fetal spinal cord. They have characterized these cells by combining several experimental approaches (FACS, immunocytochemistry, electrophysiology and RT-PCR). They then also showed positive results using these cells in transplantation studies, for the hESC line in stroke injury, and for SPC-01 in spinal cord injury (Viero et al., 2014). To accomplish this the authors have carefully analyzed major problems that are encountered in transplantation studies such as the careful characterization of the stem cells, their transplantation techniques, and their cellular reactions in grafted brain tissue. The authors have further discussed crucial matters, such as possible teratoma formation, karyotype analysis, cell viability, choice of gene expression markers for progenitor cells before and after differentiation, mycoplasma testing, and quality control of culture conditions and media. The manuscript presents a summarizing figure with the flowchart for the successful use of stem cells in the nervous system and with regenerative potentials in brain diseases. This paper builds a bridge between research scientists and clinicians to perform transplantation studies in the future. With its clarity of thoughts and clear concepts it will also no doubt lessen the confusions in the field and help to avoid inflamed debates on clinical trials, such as the Stamina one presently going on in Italy (Cattaneo and Corbellini, 2014). In this context distinguished researchers Cattaneo, Bianco, and De Luca have strongly advocated the use of rigorous scientific and medical standards for the introduction of new stem cell treatments into the clinics. For this they have recently been endowed with the Public Service Award given by the International Society for Stem Cell Research (http://www.isscr.org/home/annual-meeting/2014annualmeeting/program/2014-awards).

## Conflict of interest statement

The authors declare that the research was conducted in the absence of any commercial or financial relationships that could be construed as a potential conflict of interest.
